# Overexpression of *FLZ12* Suppresses Root Hair Development and Enhances Iron-Deficiency Tolerance in Arabidopsis

**DOI:** 10.3390/genes16040438

**Published:** 2025-04-06

**Authors:** Mingke Yan, Xin Zhang, Jinghui Gao

**Affiliations:** 1 College of Grassland Agriculture, Northwest A&F University, Yangling 712100, China; 2 College of Plant Protection, Northwest A&F University, Yangling 712100, China

**Keywords:** FCS-like zinc finger, *FLZ12*, root hair, iron deficiency, Arabidopsis

## Abstract

**Background**: The Arabidopsis FCS-LIKE ZINC FINGER (FLZ) family proteins play crucial roles in responses to various biotic and abiotic stresses, but the functions of many family members remain uncharacterized. **Methods**: In this study, we investigated the function of FLZ12, a member of the FLZ family, using a reverse genetic approach. **Results**: We found that overexpression of *FLZ12* impaired root hair development, as evidenced by marked reductions in both root hair length and number under normal growth conditions. However, deprivation of phosphate could partially restore root hair formation, although it still impeded root hair elongation. Notably, *FLZ12*-overexpressing lines exhibited greatly enhanced tolerance to iron deficiency, with seedlings exhibiting more vigorous and robust growth compared to wild-type plants. In contrast, knockout of *FLZ12* resulted in slight impact on seedling development. Further analysis revealed that FLZ12 accumulation was increased in vascular tissues of plants subjected to iron starvation, and the protein was predominantly localized within the nucleus. **Conclusions**: Integrating these findings with existing evidence, we propose that FLZ12 functions as a translational regulator through interacting with other proteins, playing dual roles in root hair development and iron-deficiency responses in *Arabidopsis*. These findings provide new insights into the FLZ-domain-containing proteins and offer molecular strategies to enhance iron uptake efficiency in crops, highlighting *FLZ12* as a promising candidate for future breeding efforts.

## 1. Introduction

Soil nutrient deficiency is one of the key environmental factors that limit plant growth. Among the essential elements needed for plant survival, phosphate (Pi) and iron (Fe) play different roles in plant development. Deficiencies in either element can disrupt metabolic processes and impair physiological functions, ultimately reducing agricultural productivity.

Phosphorus (P) is a fundamental element required by plants for various biological processes like photosynthesis and respiration. In response to Pi deficiency, plants typically accumulate anthocyanins and starches in leaves and remodel their root architecture, specifically causing the inhibition of primary root growth but the promotion of lateral root and root hair formation [1,2]. Fe, another indispensable nutrient for plants, is abundant in the Earth’s crust but often occurs in oxidized or precipitated forms, rendering it inaccessible to plants. Fe deficiency hampers root growth and chlorophyll synthesis, leading to interveinal chlorosis in young leaves and decreased efficiency of photosynthesis [3,4,5]. Consequently, both biomass accumulation and seed yield of the plant decline [6,7].

The signaling of Pi acquisition and metabolism has long been recognized as interconnected with other nutrients, particularly Fe [8,9,10]. Due to the propensity to form insoluble complexes, the antagonistic interaction between P and Fe affects plant nutrition from rhizosphere to the plant interior [11,12]. In soil, Pi naturally binds with ferric oxides (formed under aerobic conditions prevalent in most agricultural soils) to produce insoluble complexes that are inaccessible to plants’ uptake mechanisms [11]. This Fe–Pi synergy extends beyond soil chemistry to plant physiology, as evidenced by iron-dependent root growth inhibition under low-Pi conditions. It was reported that the high level of Fe accumulation in plant roots was responsible for the inhibition of primary root growth induced by Pi deficiency [13,14]. For example, A CYBDOM (CYTOCHROME B561 and DOMON DOMAIN) protein was recently found to impact Fe homeostasis and primary root growth under Pi deficiency in *Arabidopsis* [15]. Since changed Fe accumulation was observed not in the root apical meristem or elongation zone but in the maturation zone in different *Arabidopsis* lines with varied primary root length when exposed to Pi deficiency, it is still a challenge to understand the detailed molecular mechanism of how Fe accumulation in roots affects root growth under Pi deficiency [9]. Moreover, optimal Fe supply was reported to be essential for maximum root hair growth, and Fe deficiency under Pi-deficient conditions specifically impedes root hair development. [16,17]. Taken together, the tight coupling of their biochemical cycles underscores the importance of balanced nutrient management in agricultural practices.

Root hairs are single plant cell extensions of root epidermal cells. Root hair development is stimulated by various factors to increase the root surface area, thereby enhancing nutrient uptake efficiency and adaptation to soil disturbances [18,19]. These factors include nutrient perturbations such as Pi, nitrogen (N), and Fe as well as phytohormones such as auxin, ethylene, abscisic acid, and strigolactone [19,20,21]. Pi starvation leads to an increase in both root hair length and density, a process mediated by a suite of genes and proteins regulated through epigenetic, post-translational, and transcriptional mechanisms, as reviewed in [1,19,21].

By integrated analysis of several omics-based investigations of Pi-responsive genes, *At1G19200* drew our attention due to its robust transcriptional responses and putative role in membrane lipid remodeling when facing Pi deficiency [22,23,24,25]. At1G19200 belongs to the FCS-LIKE ZINC FINGER (FLZ) family proteins and was designated as FLZ12 [26]. Phylogenetic origin analysis of *Arabidopsis thaliana* FLZ proteins revealed that FLZ13, a paralog of FLZ12, displays high evolutionary similarity, with a posterior probability value higher than 0.99 [27]. The FLZ family proteins are a class of terrestrial plant-specific C2-C2 zinc finger proteins, and the expression of their encoding genes in *Arabidopsis* is differentially regulated by hormones and environmental cues [26,27,28,29]. To date, some of the members have already been proven to participate in not only various biotic or abiotic stresses but also the regulation of plant growth and development. For example, knockout of *IRM1* (*INCREASED RESISTANCE TO MYZUS PERSICAE 1*, *FLZ4*) promoted aphid population development on *Arabidopsis*, while overexpression of it obstructed aphids from reaching for the phloem and enhanced the plant resistance to the insects [30]. MARD1 (MEDIATOR OF ABA-REGULATED DORMANCY 1, FLZ9) functions downstream in the abscisic acid signaling pathway. The *mard1* seeds displayed reduced dormancy and could germinate in total darkness [31]. Reduced expression of *FLZ6* and *FLZ10* were found to negatively affect overall seedling growth, leading to reduced biomass and lateral roots and shorter primary roots under normal condition, and the impairment became more pronounced when the amount of sucrose increased [32]. More recently, FLZ13 was reported to interact with FLC (FLOWERING LOCUS C) and ABI5 (ABSCISIC ACID-INSENSITIVE 5), negatively regulating seed germination and flowering time in *Arabidopsis* [33,34]. On the other hand, the down-regulation of *FLZ6* and *FLZ10* results in enhanced expression of stress-responsive genes and improved resilience to osmotic stress during the seedling stage [35]. Most FLZ proteins were uncovered to be interaction factors of SnRK1 (Sucrose non-fermenting-1-related protein kinase), a central mediator of cellular metabolism related to energy metabolism and stress responses [28,36,37]. As representatives, FLZ6 and FLZ10 specifically interact with SnRK1α subunits in the cytoplasmic foci, functioning as repressors of SnRK1 signaling [32].

Although accumulating omics data have suggested the robust role of *FLZ12* in responses to Pi starvation, to our knowledge, no investigation has reported about its specific function and the underlying mechanisms. To unravel the mystery, in this study, we employed a reverse genetic approach to investigate *FLZ12* and demonstrated its role in regulating root hair development and seedling adaptation to Fe deficiency in *Arabidopsis*. We found that the mutation of *FLZ12* resulted in minor alteration of seedling development but the overexpression of it led to impaired root hair length and density and enhanced seedling growth under Fe deficiency. The FLZ12 protein was localized to the nucleus and likely mediates its function through interactions with binding partners to modulate protein translation.

## 2. Materials and Methods

### 2.1. Plant Materials and Growth Conditions

The wild-type *Arabidopsis* (*A. thaliana*) plants used in this study were Columbia-0 (Col-0) ecotype. The T-DNA insertion mutant *flz12-2* (SALK_062740C) was kindly provided by School of Advanced Agricultural Sciences (Peking University, Beijing, China). All the other mutants and overexpression lines were constructed in the background of Col-0. The *flz12-1* and *flz12flz13* mutants were generated by a CRISPR/Cas9 (Clustered regularly interspaced short palindromic repeats/CRISPR-associated protein 9) method following the standard procedures described in [38], and the Cas9-free T2 or higher-generation plants were used in this study.

Seeds were surface-sterilized in 75% ethanol for three minutes and in 0.5% bleach mixed with 0.5% Tween 80 for 10–13 min, followed by five rinses in sterile water. Seeds were then sown on Petri dishes containing agar media with different nutrients and stratified at 4 °C in the dark for 1–2 d. Subsequently, the Petri dishes were transferred to a growth chamber and placed vertically at 45° angle at 22–23 °C, with a photoperiod of 16 h light/8 h dark and at a light intensity of 60 µmol m^−2^ s^−1^. For the study of Pi deficiency (-P), drought, salinity stress responses, and root hair development, the SO medium described by Strieder et al. [39] was applied as the control. In phosphate-deficiency treatment, the shortage of potassium due to the absence of KH_2_PO_4_ was compensated by the addition of KCl in the same K^+^ concentration. In drought and salinity treatments, an extra 300 mM mannitol and 150 mM NaCl were added to the control media, respectively. Plants were grown in both conditions directly until the determination of each measurement. For the study of Fe-deficiency (-Fe) stress responses, MS medium with 1% (*w*/*v*) sucrose, 2.56 mM MES, and 0.8% (*w*/*v*) agar was used as the control, and Fe-EDTA was removed in the corresponding -Fe treatment. Plants were grown on MS media for the first 5 days and then transplanted onto control or -Fe plates for another 13 days until the phenotype analyses. For determination of gene expression responses to stresses, seedlings were first grown on control media for 10 days and then transferred to control, -P, or -Fe media and grown for another 3 days. The root or shoot tissues were then sampled and immediately frozen in liquid nitrogen and stored at −80 °C until RNA extraction. Three biological replicates were performed for each experiment and sampling.

### 2.2. Plasmid Constructions and Plant Transformation

To obtain the *flz12-1* and *flz12flz13* mutants, sgRNA targets of *FLZ12* and *FLZ13* were simultaneously cloned into the pHEE401 vector and transformed wild-type plants to allow a simultaneous selection of both the single- and double-gene knockout lines via CRISPR/Cas9-mediated editing [38]. For the construction of *FLZ12* overexpression (*FLZ12-OE*) lines, the CaMV 35S promoter, the genomic sequence of *FLZ12*, and the nopaline synthase (NOS) terminator were cloned and fused tandemly into the binary vector pCambia2301. To trace the tissue level expression pattern of *FLZ12*, the CaMV 35S promoter driving GUS (β-glucuronidase) on the original pCAMBIA2301 was replaced by the 1669 bp candidate promotor sequence on the upstream of *FLZ12* CDS sequence. The *Agrobacterium tumefaciens* cells carrying the constructs above were introduced into wild-type plants to obtained transformed plants as described previously [40]. For subcellular localization study of FLZ12, its CDS was C-terminally fused to the sGFP sequence under the control of the CaMV 35S promoter, and the resulting construct was cloned into the pCambia1300 multiple cloning site. Subcellular localizations of FLZ12 were examined in transient *Agrobacterium*-infiltrated leaves of *Nicotiana benthamiana* following the methods described by Contreras et al. [41].

### 2.3. Root Hair Measurement

Root hairs of 10-day-old seedlings were visualized with a Zeiss Stemi 305 dissecting microscope (Carl Zeiss Microscopy, Jena, Germany). The obtained digital images were then applied for root hair length and density determination via ImageJ (version 1.53g). Root hairs generated between the 2 and 6 mm root section from the root tip on each root were measured for determination of number and length. To minimize potential bias caused by root hair angle in length measurements, hairs exceeding the median length within each root were selectively included in the final root hair length statistics. Seven seedlings of each line were measured, and three biological repeats were performed. To present the image more clearly in this article, the raw images were processed by background subtraction and then color-inverted to generate light background using ImageJ software.

### 2.4. GUS Histochemical Analysis

Homozygous T2 transformation lines were used in the GUS staining analysis. Briefly, seedlings were vacuum-infiltrated in the GUS staining solution for 15 min and then continually incubated at 37 °C overnight. The samples were then cleared by washing using 75% ethanol several times before examination with a Zeiss Stereo Discovery V20 microscope (Carl Zeiss Microscopy, Jena, Germany). The GUS staining solution was prepared according to the recipe from [42]. More than three independent transgenic lines were tested for GUS staining, and the staining results of a representative line are displayed in the result section.

### 2.5. Subcellular Localization Analysis

The GFP fluorescence signals were examined using a LSM710 confocal microscope (Carl Zeiss Microscopy, Jena, Germany) two days after infiltration of bacterial suspension. For DAPI (4′,6-diamidino-2-phenylindole) staining, the samples were stained with 10 µg/mL DAPI solution (Solarbio, Beijing, China) for 30 min in the dark before visualization.

### 2.6. RNA Extraction and Quantitative Real-Time PCR (qRT-PCR)

Total RNA was extracted using Trizol reagent (Invitrogen, Carlsbad, CA, USA) according to the manufacturer’s instructions. The following reverse-transcription PCR and qRT-PCR were performed on a Thermo PIKOREAL 96 Real-Time PCR System (Thermo Fisher Scientific, Waltham, UK) with *TUA3* (*TUBULIN* α-*3*) used as the internal reference, as described previously [43]. The primers used in this study are listed in Table S1.

### 2.7. Statistical Analysis

Variance comparison and one-way ANOVA (analysis of variance) were performed using the R package rstatix (version 0.7.2) on evaluation of statistical differences between different samples. After ANOVA, least significant difference (LSD) multiple range tests (*p* < 0.05) via R package agricolae (version 1.3-1) were performed to compare the differences between samples. All results are presented as the mean ± SE from at least three independent biological replicates.

## 3. Results

### 3.1. Expression of FLZ12 Is Induced by Phosphate Deficiency, Drought, and Salinity

**Figure 1:**
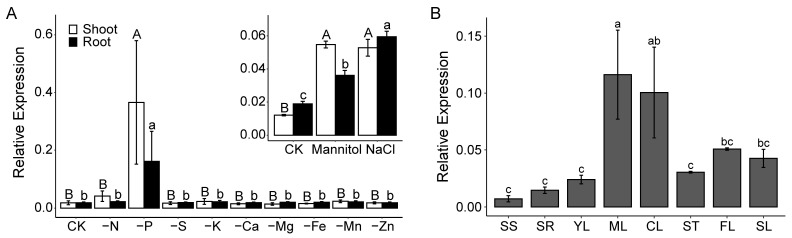
Expression patterns of FLZ12 under different abiotic stresses and in different developmental stages and tissues. (**A**) qRT-PCR analyses of the mRNA levels of FLZ12 under different abiotic stresses. Seedlings were grown on half-strength MS media for 10 days and then transplanted to control and other treatment media as indicated for another three days before sampling. (**B**) qRT-PCR analyses of the mRNA levels of FLZ12 in different developmental stages and tissues. SS, seedling shoot; SR, seedling root; YL, young rosette leaves; ML, mature rosette leaves; CL, cauline leaves; ST, stem; FL, flower; SL, flower. SS and SR are from two-week-old seedlings grown on half-strength MS media; the other samples are from plants first cultivated on half-strength MS media for 10 days and then transplanted to soil for another two weeks (YL) or four weeks (ML, CL, ST, FL, and SL). Error bars represent the means ± SE, *n* = 3. Different letters above error bars indicate significant difference (*p* < 0.05) analyzed by the least significant difference post hoc test (for A: uppercase letter, comparison in shoot; lowercase letter, comparison in root).

At the core Pi starvation-inducible genes in *Arabidopsis*, expression of *FLZ12* (*At1G19200*) was observed to be significantly and consistently up-regulated by Pi deficiency. To further explore whether FLZ12 expression changes are stress-specific or broadly responsive, we examined its transcriptional profiles across multiple stress conditions using the GENEVESTIGATOR, *Arabidopsis* eFP Browse, and Plant Regulomics databases. The results suggest that the transcription of *FLZ12* is induced not only by Pi but also drought (or osmotic stress) and salinity (Figure S1). Our localized qRT-PCR validation confirmed the expression pattern, with mRNA of *FLZ12* being induced by about 20, 4.5, and 4 times in shoots and 9, 2, and 3 times in roots by Pi deficiency, drought, and salinity, respectively (Figure 1A). Furthermore, we checked the tissue-specific expression profile of *FLZ12* and found that it was mainly expressed in mature rosette leaves and cauline leaves (Figure 1B).

### 3.2. Overexpression of FLZ12 Inhibites Root Hair Development

**Figure 2:**
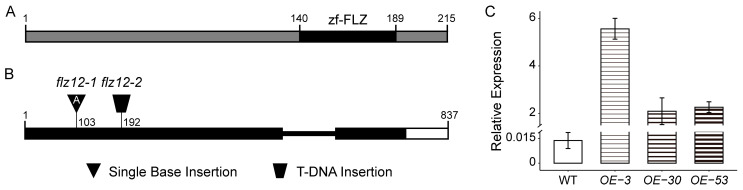
Diagram of FLZ12 structure and characterization of *flz12* mutants and overexpression lines. (**A**) Diagram of FLZ12 protein structure. (**B**) Diagram the FLZ12 gene structure and characterization of related mutants. (**C**) Validation of FLZ12 overexpression lines. Samples are shoots of 13-day-old seedlings grown on MS media.

To study whether *FLZ12* interferes in the plant responses to the above-mentioned abiotic stresses, we explored its function using a reverse genetic strategy. Two knockout mutants that interrupt the formation of the FLZ domain inside FLZ12 were obtained, namely a single base insertion mutant *flz12-1* generated by CRISPR/Cas9 method, with an adenine inserted at the first exon, and the T-DNA insertion mutant *flz12-2,* which blocks the gene structure, as shown in Figure 2B. Additionally, we generated transgenic lines overexpressing *FLZ12* (*FLZ12-OE* lines) under the control of the CaMV 35S promoter. Three independent homozygous lines, *OE-3*, *OE-30,* and *OE-53*, whose relative expression levels were significantly higher than the wild type (Figure 2C), were selected for subsequent studies.

**Figure 3:**
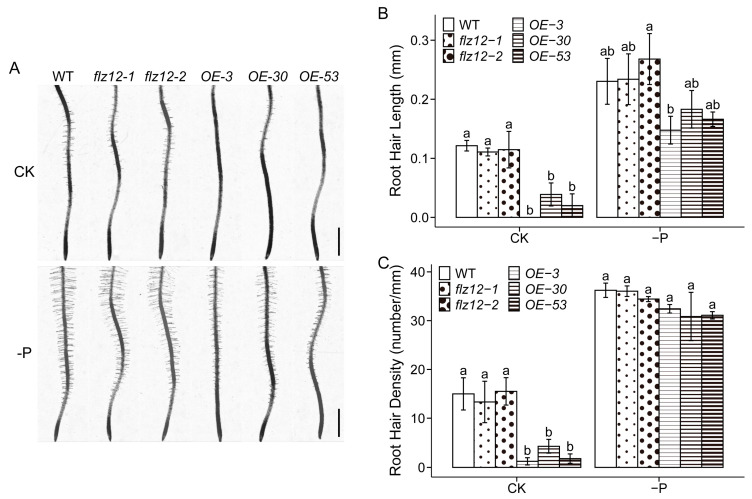
Overexpression of FLZ12 represses root hair development. Root hair phenotype (**A**), length (**B**), and density (**C**) of wild type (WT), *flz12,* and *FLZ12-OE* seedlings grown under normal (CK) and Pi deficiency (-P) conditions for 10 days. Scale bar, 1 mm. Error bars represent the means ± SE, *n* = 3. Different letters above error bars indicate significant difference (*p* < 0.05) analyzed by the least significant difference post hoc test, and comparisons were conducted within treatment.

Based on FLZ12’s stress-responsive expression profile, we analyzed the phenotypic responses of different lines grown in the normal condition and Pi deficiency, drought, and salinity treatments. Unfortunately, in general, we observed no distinct shoot abnormalities upon all the lines under drought or salinity conditions. Moreover, although we detected high transcript levels of *FLZ12* in mature rosette leaves and cauline leaves, no discernible phenotypic alterations was found from the expression-interfered plants after being transplanted to soil at ten days after the start of germination (DAG). While different from shoots, the *FLZ12-OE* lines exhibited defects in root hair development (Figure 3). Both root hair length and density were substantially decreased in *FLZ12-OE* lines compared with the wild type in normal condition. When Pi was deprived, the root hair length of the *FLZ12-OE* lines was significantly curtailed, while the root hair density was merely slightly declined and statistically comparable with the wild type. Furthermore, the severity of root hair suppression was negatively correlated with *FLZ12* transcript abundance, suggesting a dose-dependent inhibitory effect. Conversely, *flz12* displayed no noticeable disturbance in the root hair growth (Figure 3). Taken together, overexpression of *FLZ12* could suppress root hair formation and elongation, while the hair formation could be restored by a shortage of Pi supply.

### 3.3. Overexpression of FLZ12 Enhances Seedling Tolerance to Iron Deficiency

**Figure 4:**
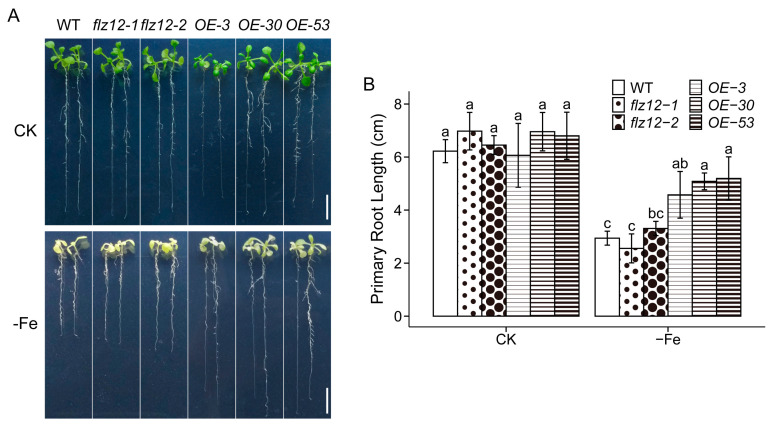
Overexpression of FLZ12 enhances seedling growth under iron-deficiency condition. Phenotype (**A**) and primary root length (**B**) of wild type (WT), *flz12,* and *FLZ12-OE* seedlings grown under normal (CK) and Fe-deficiency (-Fe) conditions. Seedlings were grown in normal condition for 5 days before being transferred to CK or -Fe media for another 13 days. Scale bar, 1 cm. Error bars represent the means ± SE, *n* = 3. Different letters above error bars indicate significant difference (*p* < 0.05) analyzed by the least significant difference post hoc test, and comparisons were conducted within treatment.

Taking into consideration that there is broad crosstalk between Pi and Fe both in nutrients and their signaling in plants, we further tested the possible involvement of *FLZ12* under Fe deficiency. Interestingly, *FLZ12-OE* lines were significantly more tolerant to Fe shortage than the wild type and knockout mutants (Figure 4 and Figure S2). Overall, when Fe was deficient, the *FLZ12-OE* plants were much stronger, characterized by more, larger, and greener leaves; the root systems were more developed, and the primary root grew much faster. However, unexpectedly, no visible defect or modification was observed in the knockout mutants (Figure 4).

### 3.4. FLZ12 Displays Little Duplicate Effect with FLZ13

One of the major reasons of failure in detecting a visible mutant phenotype for some genes is the existence of functional redundant partners. For *flz12*, we speculate that this may be due to a redundant effect of FLZ13. To test this, a double-mutant *flz12flz13* was generated using the methods of CRISPR/Cas9. However, no significant difference in phenotype was observed between the *flz12flz13* and wild type in the conditions tested above (Figure S3), indicating that there are other duplicate proteins, or they are functionally irredundant.

### 3.5. FLZ12 Mainly Expresses in the Vasculature and the Protein Localizes in the Nucleus of the Cell

**Figure 5:**
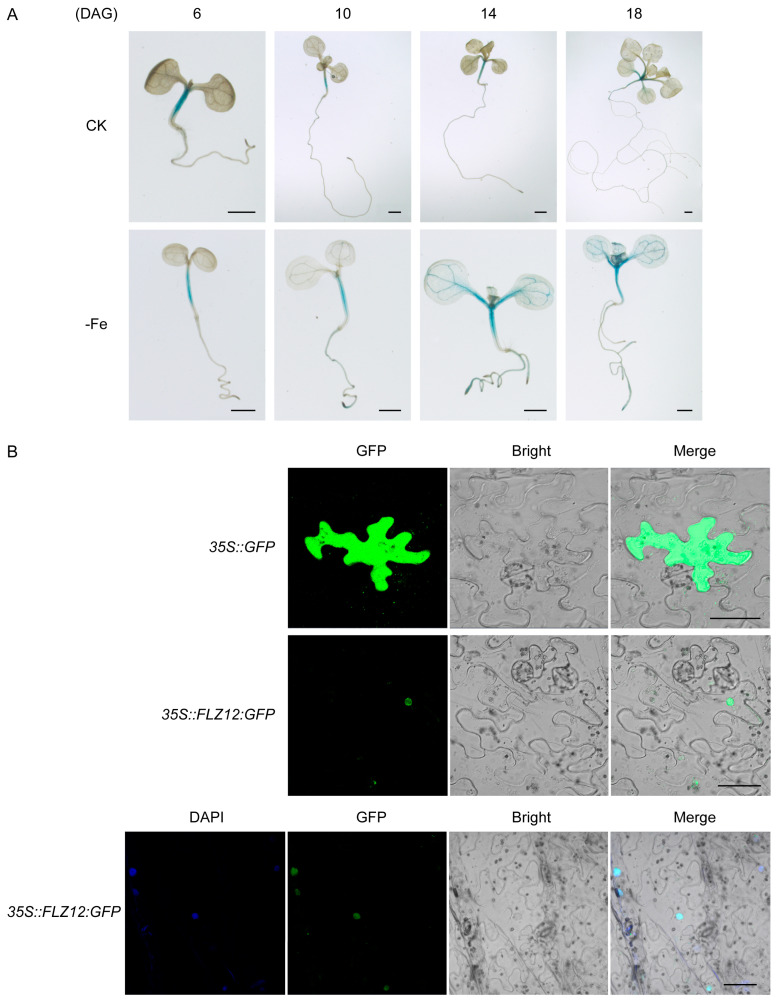
Expression pattern and subcellular localization of FLZ12. (**A**) Expression pattern of *PRO^FLZ12^*::*GUS* in transformed *Arabidopsis* plants. Seedlings were grown on normal (CK) and Fe-deficiency (-Fe) conditions for 6, 10, 14, and 18 days, respectively. DAG, days after the start of germination. Scale bar, 1 cm. (**B**) Subcellular localization of 35S::FLZ12::GFP in *N. benthamiana* leaves. Scale bar, 50 µm. DAPI, 4′,6-diamidino-2-phenylindole; GFP, green fluorescent protein.

The fascinating behavior of *FLZ12-OE* lines under iron-deficiency stress indicates a promising application prospect. To better understand the function mechanism, we tried to uncover the detailed responsive profile of *FLZ12* against Fe deficiency. Because the overall expression of *FLZ12* in roots or shoots is not changed under Fe-starvation stress (Figure 1A), we speculate that at the sub-organ level, namely at tissue or cell levels, it may have altered. To clarify this possibility, the promoter sequence of *FLZ12* was fused to a GUS reporter gene (*PRO^FLZ12^*:*GUS*) and transformed into wild-type plants. The GUS activity was then detected in seedlings grown under normal or Fe-deficiency conditions. GUS profiling revealed that under both conditions, *FLZ12* was strongly expressed in the hypocotyls of plants, especially in the steles. As plants reached 14 DAG and beyond, the signals gradually expanded to the vascular tissues of leaves and roots, with those in the veins in the cotyledon being particularly intensive. In contrast to control, this trend was more pronounced when Fe was deficient, and with the stress prolonged, the GUS activities became more intensified and could be clearly identified in the steles of roots (Figure 5A). These data indicate a role of *FLZ12* in Fe-deficiency responses, although the lack of it resulted in no apparent disturbance of plant growth.

To investigate the subcellular distribution of FLZ12 protein, we fused GFP to the C-terminal of FLZ12 under the control of CaMV 35S promoter and transiently expressed the GFP-fusion proteins in tobacco leaves. Similar to other FLZ domain-containing proteins, GFP fluorescence was confined to the nucleus of the cell, and this could be further evidenced by the colocalization with nuclear counterstain DAPI (Figure 5B).

## 4. Discussion

### 4.1. Uncertain Disturbances in Seedling Growth Caused by Overexpression of FLZ12

In this study, *FLZ12* was found to be highly expressed in mature rosette leaves and cauline leaves of *Arabidopsis*. Although we maintained the environmental conditions as consistently as possible during each plant cultivation, and generally no consistent phenotypical difference was detected between the wild-type and the homozygous genetically modified plants, we observed disturbances in the growth of several *FLZ12-OE* lines (about 1/3 of all the independent *FLZ12-OE* lines) several times when growing in normal soil. The plants developed dark green rosette leaves and bolted later than the wild type. This was reminiscent of the phenotype of *IRM1* (*FLZ4*) and *FLZ13*. The knockout mutant of *IRM1* is morphologically similar to the wild type, while the overexpression lines had dark-green and smaller rosette leaves, delayed bolting time, and smaller size of flowers and siliques [30]. In addition, *FLZ13-OE* plants bolted and flowered significantly later, while *flz13* plants behaved opposite to the wild type [33]. However, all the *FLZ12* lines grew similar to the wild type more often. We have not detected what stimulus triggered these abnormalities.

### 4.2. Negative Role of FLZ12 in Root Hair Development and Its Link with SnRK1 and PLDζ2 (PHOSPHOLIPASE Dζ2)

In the present study, there was a decrease in root hair density and length of *FLZ12-OE* lines under either Pi sufficiency or deficiency conditions, and the suppressing effect was more pronounced when Pi was sufficient (Figure 3). That is to say, the Pi deficiency signal can compensate for the FLZ12-induced root hair defect, especially root hair emergence. A comprehensive examination and comparison of FLZ- and Pi-induced transcriptome variation would help in finding the signal joint [1]. The root hair phenotypes of FLZ12 were reminiscent of the root hair behavior of KIN10 (SNF1-RELATED PROTEIN KINASE 1.1)-related lines [44]. KIN10 is one of the α-catalytic subunits of SnRK1, a central mediator of energy signaling [36]. FLZ12 was reported to directly interact with KIN10 when expressed in yeast [28]. Overexpression of *KIN10* resulted in significantly decreased root hair number and length in normal conditions, while in RNAi-plants, the root hairs developed roughly similarly to that of the wild type [44]. Given that both FLZ12 and KIN10 possess several homologs in the *Arabidopsis* genome, it is possible that functional redundant paralogs might have compensated for the single gene losses. However, simultaneous knockout of *flz12* and its phylogenetic closest homolog *flz13* displayed no visible effect compared with the wild type (Figure S3A–C). The potential reason may be that there are still other functional, redundant proteins of FLZ12, alternative pathways could completely compensate for the defect of FLZ12, or it is not FLZ12 itself controlling root hair development in the wild type but instead its ectopic expression. Previous studies have suggested that FLZ12 might participate in membrane lipid remodeling under phosphate-deficiency stress, and it was closely linked with PLDζ2 (PHOSPHOLIPASE Dζ2), a member of phospholipase D proteins [22,45]. PLDζ2 has a positive effect on primary root growth but is a negative regulator of both root hair density and length under Pi-deficiency conditions [46,47]. When Pi was withheld, the root hair development defects were partially alleviated, and hair density was only marginally reduced compared to the control. This conditional phenotypic rescue suggests a genetic antagonism between FLZ12 and PLDζ2, potentially converging on phospholipid signaling pathways to modulate phosphate homeostasis and root epidermal patterning.

### 4.3. FLZ12 Is a Promising Candidate in Improving Plant Iron-Deficiency Resilience via a FIT-IRT1-Independent Pathway

Many reports have confirmed the pervasive nutrient crosstalk between Pi and Fe [8,9,10,48]. Besides Pi, we also tested the responses of FLZ12 against Fe deprivation and found that stunted seedling growth under Fe deficiency was greatly mitigated in *FLZ12-OE* lines compared with the wild type (Figure 4). It can be concluded that the *FLZ12-OE* lines could positively adapt to a Fe-deficiency signal, serving as a promising candidate in future breeding work towards crops with higher Fe efficiency. Although the loss of FLZ12 resulted in a negligible impact on seedling growth, the enhanced expression of *FLZ12* in the vascular tissue of hypocotyls and cotyledons by Fe deprivation demonstrates that FLZ12 does play a role in Fe starvation responses in Arabidopsis. As far as we know, there is no report about the direct involvement of PLDζ2, the putative functional partner of FLZ12, in Fe signaling and responses. Nevertheless, as an interaction factor of FLZ12, SnRK1 has a profound impact on genes related to iron acquisition and homeostasis, with iron metabolism genes being strongly down-regulated in its loss-of-function mutant [49]. Moreover, the Fe-efficient genotype was reported to be able to induce energy-controlling pathways possibly regulated by GmSnRK1 to promote nutrient recycling and stress responses in iron-deficient conditions in soybean [50]. SnRK1 kinases are key regulators in keeping plant energy balance and homeostasis and can be activated by abiotic stresses that cause energy deficits by affecting photosynthesis [51]. Since Fe is essential for the structure and/or function of the photosynthetic electron transfer chain, Fe deficiency could impair the photosynthetic apparatus and remodel the signaling pathway of SnRK1 [6]. Given these results, we propose that FLZ12 participates in this Fe-deficiency-induced remodeling of energy homeostasis, and future exploration of the functional mechanism of FLZ12 on Fe deficiency can focus on the FLZ12–SnRK1 interaction module. In the present study, FLZ12 was found to localize in the nuclei of cells (Figure 5), and this was in line with most FLZ proteins described in previous publications [26,28]. However, another study revealed that FLZ proteins act as an SnRK1 scaffold in land plants, and the interaction of FLZ with SnRK1 colocalizes to the endoplasmic reticulum. Based on this, the FLZ proteins are supposed to be responsible for the recruitment of SnRK1 to participate in the regulation of protein translation [51,52]. Integrating these findings, we propose that FLZ12 functions as a connective component in Fe-starvation signal transduction to modulate downstream protein translation, but its functioning subcellular localization needs to be further clarified. Actually, we tested the mRNA level of several representative Fe-deprivation-related genes, from transcription factors *FIT* (*FE-DEFICIENCY INDUCED TRANSCRIPTION FACTOR 1*) and *bHLH38*/*39* (*BASIC HELIX-LOOP-HELIX 38/39*) to a ferric reductase oxidase enzyme *FRO2* (*FERRIC REDUCTION OXIDASE 2*), a high-affinity iron uptake transporter *IRT1* (*IRON-REGULATED TRANSPORTER 1*) and a Fe-nicotianamine translocation transporter *YSL2* (*YELLOW STRIPE LIKE 2*), and ferritins *FER1*/*4* (*FERRETIN 1/4*). Unfortunately, none of these genes displayed consistent transcriptional alteration in responses to *FLZ12* expression. Therefore, we hypothesize that at the protein abundance level, some of the Fe-responding components may have changed. Given their similarity to the root hair phenotype, the *flz12* or *flz12flz13* mutants exhibited no visible difference with wild type; we assume that overexpression of *FLZ12* positively regulates Fe-deficiency responses in *Arabidopsis* but likely in a FIT-IRT1-independent manner and functions together with other unknown components. Notwithstanding, the other possibilities, such as the existence of redundant components, can currently not be excluded. The absence of fitness costs or growth penalties in *FLZ12-OE* lines growing in normal conditions, combined with their enhanced Fe-deficiency tolerance, positions FLZ12 as a promising candidate for molecular breeding aimed at improving crop Fe-deficiency adaptation.

## 5. Conclusions

In conclusion, our findings demonstrate that overexpression of *FLZ12* suppresses root hair development in *Arabidopsis* but promotes seedling growth and development under Fe-starvation stress. This fine performance under Fe deficiency particularly implicates the high value of employing FLZ12 in breeding new Fe-smart crops. The localization of FLZ12 and some related studies have indicated that FLZ12 may function as a scaffold protein regulating translation, and we suggest that there may be other interactive partners or genetic redundant components of *FLZ12* that exist in the genome. Future work to elucidate the interaction factors and characterize the putative protein redundancy would help clarify the detailed mechanism of how FLZ12 connects these abiotic stress responses and downstream molecular alterations.

## Data Availability

The original contributions presented in this study are included in the article/Supplementary Material. Further inquiries can be directed to the corresponding author(s).

## Supplementary Materials

The following supporting information can be downloaded at https://www.mdpi.com/article/10.3390/genes16040438/s1. Figure S1: Impact of different perturbations on FLZ12 expression level. Figure S2: Biomass of FLZ12-OE lines subjected to Fe deficiency. Figure S3: Phenotype analysis of double mutant *flz12flz13*. Table S1: Primers used in this study.

## Abbreviations

The following abbreviations are used in this manuscript:

ABI5	ABSCISIC ACID-INSENSITIVE 5
ANOVA	Analysis of variance
bHLH	BASIC HELIX-LOOP-HELIX
CRISPR/Cas9	Clustered regularly interspaced short palindromic repeats/CRISPR-associated protein 9
CYBDOM	CYTOCHROME B561 AND DOMON DOMAIN
DAG	Days after the start of germination
DAPI	4′,6-Diamidino-2-phenylindole
Fe	Iron
-Fe	Iron deficiency
FER	FERRETIN
FIT	FE-DEFICIENCY INDUCED TRANSCRIPTION FACTOR 1
FLC	FLOWERING LOCUS C
FLZ	FCS-LIKE ZINC FINGER
FRO2	FERRIC REDUCTION OXIDASE 2
IRM1	INCREASED RESISTANCE TO MYZUS PERSICAE 1
IRT1	IRON-REGULATED TRANSPORTER 1
KIN10	SNF1-RELATED PROTEIN KINASE 1.1
MARD1	MEDIATOR OF ABA-REGULATED DORMANCY 1
OE	Overexpression
P	Phosphorus
-P	Phosphate deficiency
Pi	Phosphate
PLDζ2	PHOSPHOLIPASE Dζ2
qRT-PCR	Quantitative real-time PCR
SnRK1	Sucrose non-fermenting-1-related protein kinase
TUA3	TUBULIN α-3
YSL2	YELLOW STRIPE LIKE 2
