# Supplementary material for: Overexpression of FLZ12 Suppresses Root Hair Development and Enhances Iron-Deficiency Tolerance in Arabidopsis
Source: Genes (Basel). 2025 Apr 6;16(4):438. doi: 10.3390/genes16040438 (PMC12027241; doi:10.3390/genes16040438)
Supplement: Supplementary file 1 [file genes-16-00438-s001.zip › genes-3529170-supplementary.pdf]

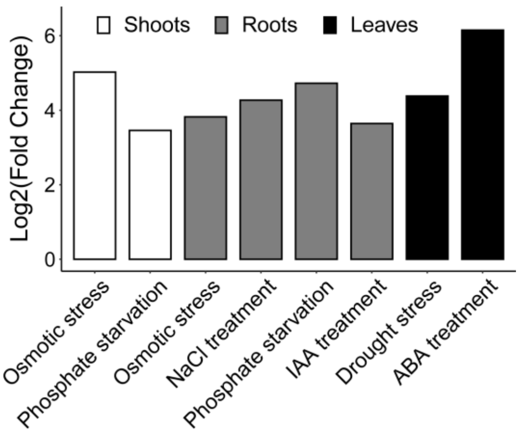

Figure S1. Impact of different perturbations on FLZ12 expression level. Expression data was exported from the Plant Regulomics database and representative data was manually selected.

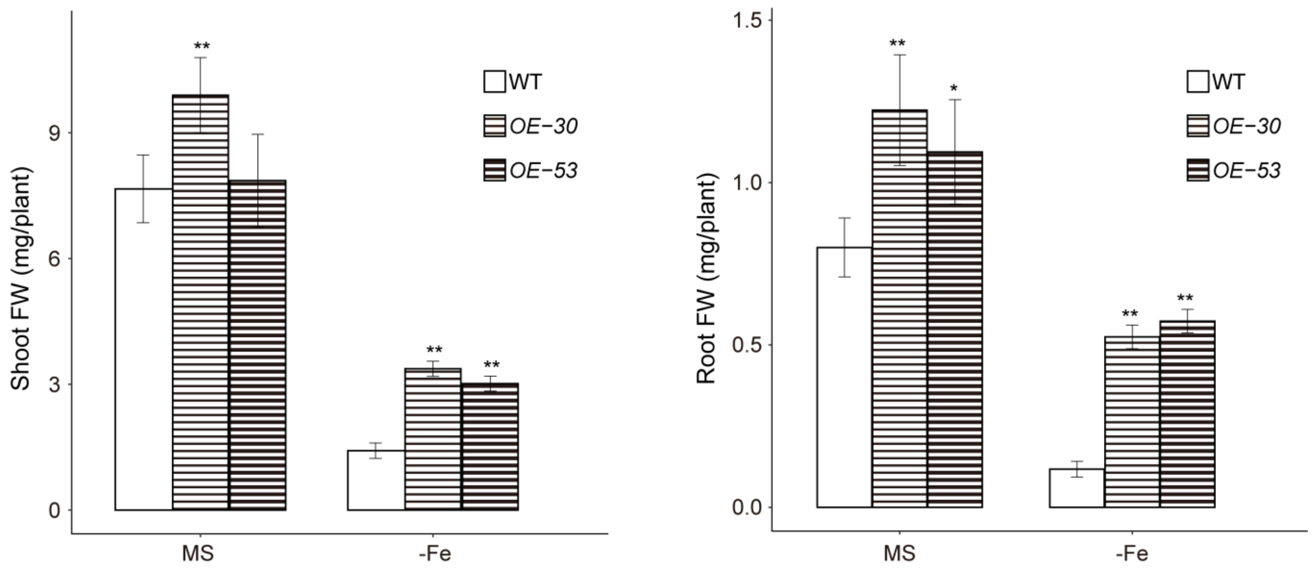

Figure S2. Biomass of *FLZ12-OE* lines subjected to Fe deficiency. Fresh weight of wild type (WT) and *FLZ12-OE* seedlings grown under normal (CK) and Fe deficiency (-Fe) conditions for 18 d. Error bars represent the means ± SE, $n = 3$. Asterisk above error bars indicate significant difference (*: $p < 0.05$; **: $p < 0.01$) analyzed by student's t-test, and comparisons were conducted between individual OE lines with WT under each cultivation condition.

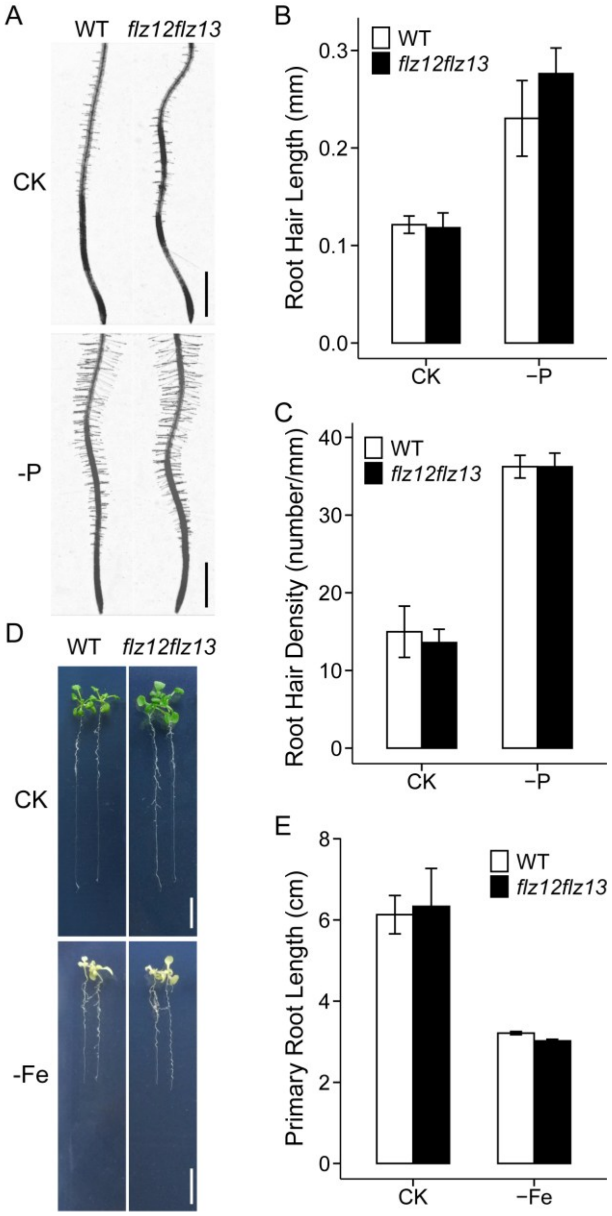

Figure S3. Phenotype analysis of double mutant *flz12flz13*. A-C, Root hair phenotype, length and density of wild type (WT) and *flz12flz13* seedlings grown under normal (CK) and Pi deficiency (-P) conditions for 10 days. Scale bar, 1 mm. D and E, Phenotype and primary root length of wild type (WT) and *flz12flz13* seedlings grown under normal (CK) and Fe deficiency (-Fe) conditions. Seedlings were grown in normal condition for 5 days before transferring to CK or -Fe media for another 13 days. Scale bar, 1 cm. Error bars represent the means ± SE, $n = 3$.

Table S1. Primers used in this study

Primer name	Sequece (5' to 3')	Usage
FLZ12-OE-F	GCGGTCTAGAAGCAATCCAATGACCTG	Genomic sequence cloning for overexpression
FLZ12-OE-R	TCCACTGCAGCATAGAAAACTCAAAACGAAG	Genomic sequence cloning for overexpression
FLZ12-GUS-F	GGGGTACCGAAGGGTCGTCTTCTTCGATGC	Promotor cloning for fusing with GUS
FLZ12-GUS-R	CATGCCATGGGTGGGAGAGTTTCCAGGTCATTG	Promotor cloning for fusing with GUS
FLZ12-GFP-F	GGGGTACCATGGTGGTTCCAGGTAAAAACTCC	CDS cloning for fusing with GFP
FLZ12-GFP-R	GCTCTAGATATAAGGAATATTCCGGGGGAGGC	CDS cloning for fusing with GFP
FLZ12-q-F	GAAATCGCCGAGGAGTCAC	RT-qPCR
FLZ12-q-R	CTCTTTACTACAAAATCCTTCATCTCC	RT-qPCR
TUA3-q-F	GTGCTGAAGGTGGAGACGAT	RT-qPCR
TUA3-q-R	AACACGAAGACCGAACGAAT	RT-qPCR
FLZ12-Cas-BsF	ATATATGGTCTCGATTGCGTCGCCTG-GAAGTTCGAAGGTT	CRSPR/Cas9
FLZ12-Cas-F0	TGCGTCGCCTGGAAGTTCGAAGGTTTTA-GAGCTAGAAATAGC	CRSPR/Cas9
FLZ13-Cas-R0	AACCGGTTCACTCGAA-GCGGTTCCAATCTCTTAGTCGACTCTAC	CRSPR/Cas9
FLZ13-Cas-BsR	ATTATTGGTCTCGAAACCGGTTCACTCGAA-GCGGTTCCAA	CRSPR/Cas9
